# 伴ASXL1基因突变初诊急性髓系白血病患者的临床特征及生存分析

**DOI:** 10.3760/cma.j.issn.0253-2727.2022.10.006

**Published:** 2022-10

**Authors:** 闻博 贾, 金婷 刘, 新雨 杨, 汉阳 吴, 义洪 魏, 灿 灿, 锐卿 王, 娜 何, 朝阳 谷, 道新 马, 春岩 纪

**Affiliations:** 山东大学齐鲁医院血液科，济南 250012 Department of Hematology, Qilu Hospital, Shandong University, Jinan 250012, China

**Keywords:** 白血病，髓系，急性, 基因，ASXL1, DNA突变分析, 生存分析, Leukemia, myeloid, acute, Gene, ASXL1, DNA mutational analysis, Survival analysis

## Abstract

**目的:**

研究伴ASXL1基因突变初诊急性髓系白血病（AML）患者的临床特征及生存。

**方法:**

对2016年1月至2021年4月就诊于山东大学齐鲁医院的初诊非M_3_型AML患者的临床资料进行回顾性研究，分析ASXL1突变阳性患者的临床特征及生存。基因突变检测采用二代测序法。

**结果:**

①初诊且资料完整的256例AML患者纳入研究，其中ASXL1突变阳性（ASXL1^+^）47例，阴性（ASXL1^−^）209例。将所有患者分为老年组（≥60岁）92例、中年组（45～59岁）92例和青年组（≤44岁）72例。②与ASXL1^−^患者相比，ASXL1^+^患者年龄大、WBC高、首疗程完全缓解（CR_1_）率低（*P*值均<0.05）。老年组ASXL1^+^患者WBC、异常细胞占有核细胞比例高于ASXL1^−^患者（*P*值均<0.05）；青年组ASXL1^+^患者WBC高于ASXL1^−^患者（*z*＝−2.314，*P*＝0.021）。③ASXL1突变与IDH2突变相关（*P*＝0.018，*r*＝0.34）。在ASXL1^+^患者中，高变异等位基因频率（VAF）组（VAF>40％）外周血原始幼稚细胞比例高于低VAF组（VAF<20％），且碱基重复和替换突变患者的异常细胞占有核细胞比例高于缺失突变患者（*P*值均<0.05）。④ASXL1^+^患者中位总生存（OS）时间和无进展生存（PFS）时间均短于ASXL1^−^患者（10个月对20个月，10个月对 17个月；*P*值均<0.05）。多因素分析示异常细胞占有核细胞比例≥20％、复杂核型、TET2突变均为影响ASXL1^+^患者预后的独立危险因素（*P*值均<0.05）。

**结论:**

伴ASXL1突变非M_3_型AML患者初诊时WBC、异常细胞占有核细胞比例均高，CR_1_率低，OS及PFS时间短。ASXL1突变患者中高VAF、碱基重复和替换突变与预后不良有关，异常细胞占有核细胞比例高、复杂核型和TET2突变均为影响预后的独立危险因素。

## Abstract (en)

**Objective:**

To examine the survival rates and clinical characteristics of people with newly discovered non-M_3_ acute myeloid leukemia（AML）who carry the ASXL1 gene mutation.

**Methods:**

From January 2016 to April 2021, the clinical information of patients with newly diagnosed non-M_3_ AML at Shandong University's Qilu Hospital was retrospectively examined, and their clinical characteristics and survival were compared and analyzed. Gene mutation was detected by next-generation sequencing.

**Results:**

① The study included 256 AML patients who were initially diagnosed and had complete data, including 47 cases of ASXL1 gene mutation-positive（ASXL1^+^）patients and 209 cases of ASXL1 gene mutation-negative（ASXL1^−^）patients. All patients were divided into three groups: elderly（≥60 years old, *n*＝92）, middle-aged（45–59 years old, *n*＝92）, and young（≤44 years old, *n*＝72）. ②WBC, and age were higher in patients with ASXL1 mutations compared to ASXL1^−^ patients, while complete response after the first round of treatment（CR_1_）was lower（*P*<0.05）. In the elderly group, WBC and the proportion of aberrant cells in nuclear cells in ASXL1^+^ patients were higher than those in ASXL1^−^ patients（*P*<0.05）. In the young group, the WBC of ASXL1^+^ patients was higher than that of ASXL1^−^ patients（*z*＝−2.314，*P*＝0.021）. ③IDH2 mutation and ASXL1 mutation was related（*P*＝0.018, *r*＝0.34）. In ASXL1^+^ patients, the proportion of peripheral blasts in the high VAF group（VAF>40％）was higher than that in the low VAF group（VAF<20％）, and the proportion of aberrant nuclear cells was higher in the duplication and replacement mutation patients than in the deletion mutation patients（*P*<0.05）. ④The overall survival（OS）and progression-free survival（PFS）of ASXL1^+^ patients were shorter than those of ASXL1^−^ patients（median, 10 months *vs* 20 months，10 months *vs* 17 months; *P*<0.05）. The proportion number of aberrant cells in nuclear cells（≥20％）, complex karyotypes, and TET2 mutation were all independent risk variables that had an impact on the prognosis of ASXL1^+^ patients, according to multivariate analysis（*P*<0.05）.

**Conclusion:**

ASXL1-mutated non-M_3_ AML patients have higher WBC in peripheral blood, a higher proportion of aberrant cells in nuclear cells, lower CR_1_ rate, and shorter OS and PFS. Additionally, a poor prognosis is linked to higher VAF, duplication, and substitution mutations in the ASXL1 gene, as well as the high proportion of aberrant cells in nuclear cells, complex karyotype, and TET2 mutation.

急性髓系白血病（AML）是一种常见的恶性血液病，AML患者常伴有多种基因突变，ASXL1基因为其中之一。ASXL1是Trithorax和Polycomb家族的增强子，定位于染色体20q11，它编码一个长为1541个氨基酸的核蛋白，该蛋白是多梳蛋白族的一员，也是维持稳态和其他基因座稳定所必需的[1]–[2]。已有研究表明，ASXL1基因突变与原发性骨髓纤维化（PMF）[3]、慢性嗜酸性粒细胞白血病（CEL）[4]、慢性中性粒细胞白血病（CNL）[5]、造血干细胞移植后供者源白血病（DCL）[6]有关。在本研究中，我们通过分析我院接受二代测序（NGS）检测的初诊非M_3_型AML患者的临床特征及预后，评价基于NGS检测技术下克隆性基因突变的预后价值，以期更好地协助诊治、指导临床决策。

## 病例与方法

1. 病例资料：选取2016年1月至2021年4月就诊于山东大学齐鲁医院血液科且接受NGS检测的656例非M_3_型AML患者，ASXL1突变率为11.1％。选取其中初诊、非M_3_、接受NGS检测和临床资料完整的256例患者进行回顾性研究。诊断均符合《中国成人急性髓系白血病（非急性早幼粒细胞白血病）诊疗指南（2021年版）》诊断标准[7]。按年龄分为老年（≥60岁）（92例）、中年（45～59岁）（92例）、青年（≤44岁）（72例）三组。分析患者的一般临床特征、实验室检查和随访结果等临床资料。

2. NGS及微小残留病（MRD）检测方法：基因突变采用二代测序法进行检测，测序仪器采用Illumina MiSeqDx。检测基因包括：AML1、ASXL1、BCOR、CALR、CBL、CEBPA、DNMT3A、ETV6、EZH2、FLT3-ITD、FLT3-TKD、GATA2、IDH1、IDH2、JAK2、KIT、KRAS、NF1、NPM1、NRAS、PHF6、RUNX1、SETBP1、SF3B1、SH2B3、SRSF2、STAG2、STAT3、TET2、TP53、U2AF1、WT1、ZRSR2等。基因检测结果以定性（阳性、阴性）、定量（变异等位基因频率，VAF）和突变位点进行表示。MRD水平通过流式细胞术进行检测。以上检测由山东大学齐鲁医院血液病研究室完成。

3. 治疗：256例患者中，除27例患者未接受化疗外，其余患者入院后均行诱导治疗。中年及青年患者诱导方案包括：标准剂量阿糖胞苷（Ara-C）联合去甲氧柔红霉素（IDA）（IA方案）或柔红霉素（DNR）（DA方案）的常规诱导方案以及包含高三尖杉酯碱（HHT）联合标准剂量Ara-C（HA）的其他诱导方案[7]。老年患者应用低强度化疗方案：地西他滨；小剂量化疗±G-CSF（如小剂量Ara-C为基础的CAG、CHG、CMG等方案，C：Ara-C；A：阿克拉霉素；H：HHT；M：米托蒽醌）；地西他滨联合小剂量化疗，包括地西他滨+CAG、地西他滨+小剂量Ara-C方案等[7]。老年、中年、青年组患者中，ASXL1突变阳性（ASXL1^+^）和ASXL1突变阴性（ASXL1^−^）患者的诱导化疗方案差异无统计学意义（*P*>0.05）。

4. 随访及定义指标：随访截止时间为2021年12月5日，随访资料来源于门诊病历、住院病历、电话随访记录等。所有患者的中位随访时间为38（1～66）个月，失访患者25例（9.8％）。老年组中位随访时间为30（1～66）个月，失访患者12例（13.0％）；中年组中位随访时间为39（1～52）个月，失访患者13例（14.1％）；青年组中位随访时间为41（1～55）个月，无失访患者。总生存（OS）时间定义为首次确诊至死亡或随访截止的时间[8]，无进展生存（PFS）时间定义为首次确诊至疾病复发、进展、死亡或随访截止的时间[8]。

5. 统计学处理：采用SPSS 22.0和R 4.1.0软件进行统计学分析。计量资料以中位数（范围）表示，采用Mann-Witney *U*检验进行比较。分类资料以例数（构成比）表示，采用卡方检验进行比较，并采用Kaplan-Meier法描绘生存曲线。采用Cox比例风险回归模型进行预后因素分析。*P*<0.05为差异有统计学意义。

6. 生信分析处理：采用Alphafold2和Chimerax软件对蛋白结构进行预测及可视化，采用Chimera软件、Mutation Taster网站进行蛋白结构比较及突变致病性、保守性分析。

## 结果

1. AML患者一般临床特征：256例AML患者中男130例，女126例，中位年龄53（14～87）岁。初诊中位WBC 3.5（0.08～479.73）×10^9^/L，HGB 66.5（7～148）g/L，PLT 37.5（0～483）×10^9^/L，骨髓原始细胞比例为55％（0～98％），外周血原始幼稚细胞比例为34％（0～98％），异常细胞占有核细胞比例为34％（0～96％）。FAB分型：M_0_ 0例，M_1_ 5例，M_2_ 7例，M_4_ 71例，M_5_ 147例，M_6_ 1例，未分类 25例。根据细胞遗传学/分子遗传学指标危险度分级标准[7]，70例为预后良好，124例为预后中等，62例为预后不良。

2. ASXL1突变的临床相关性：256例患者中，ASXL1^+^患者较ASXL1^−^患者年龄大、初诊时WBC高、首疗程完全缓解（CR_1_）率低（*P*值分别为0.004、0.002、0.001）（表1）。进一步按年龄分层进行比较，老年、中年和青年组中ASXL1^+^和ASXL1^−^患者性别、PLT、FAB分型差异均无统计学意义（*P*值均>0.05）。老年组中ASXL1^+^患者初诊时中位WBC、异常细胞占有核细胞比例均显著高于ASXL1^−^患者（17.67×10^9^/L对14.17×10^9^/L，*P*＝0.031；38％对20％，*P*＝0.022），青年组中ASXL1^+^患者初诊时中位WBC高于ASXL1^−^患者（49.44×10^9^/L对17.78×10^9^/L，*P*＝0.021）。ASXL1突变对中、青年组患者CR_1_率有显著影响，中年和青年组中ASXL1^+^患者CR_1_率均显著低于ASXL1^−^患者［28.6％（4/14）对63.2％（36/57），*P*＝0.019；25.0％（1/4）对72.4％（42/58），*P*＝0.047］。

**表1:** ASXL1基因突变阳性和阴性急性髓系白血病临床特征比较

临床特征	ASXL1^+^组（47例）	ASXL1^−^组（209例）	统计量	*P*值
性别（例，男/女）	28/19	102/107	*χ*^2^=1.781	0.182
年龄[岁，M(范围)]	55(14~85)	51(14~87)	*z*=−2.843	0.004
WBC[×10^9^/L，*M*(范围)]	5.2(0.59~271.47)	1.8(0.08~479.73)	*z*=−3.147	0.002
HGB[g/L，*M*(范围)]	68(21~135)	65(7~148)	*z*=−1.431	0.152
PLT[×10^9^/L，*M*(范围)]	46(2~298)	29(0~483)	*z*=−1.341	0.180
FAB分型(例,M_0_/M_1_/M_2_/M_4_/M_5_/M_6_/未分类)	0/0/2/8/29/1/7	0/5/5/63/118/0/18		–
外周血原始幼稚细胞比例[％，*M*(范围)]	26(0~89)	42(0~98)	*z*=−1.246	0.213
骨髓原始细胞比例[％，*M*(范围)]	45(0~95)	64(0~98)	*z*=−1.682	0.092
异常细胞占有核细胞比例[％，*M*(范围)]	28(5~88)	39(0~96)	*z*=−1.594	0.111
首疗程化疗后MRD[％，*M*(范围)]	2.62（0~77.80）	0.61（0~73.26）	*z*=−1.930	0.054
CR_1_率[CR_1_患者/评估患者（％)]	8/29（27.0）	95/152(60.5)	*χ*^2^=12.105	0.001

注：MRD：微小残留病；CR_1_：首次完全缓解

3. ASXL1的基因突变特征分析：256例AML患者中共47例（18.4％）伴有ASXL1基因突变，其中伴ASXL1 12号外显子突变患者46例，伴8号外显子突变患者1例。将12号外显子突变的46例患者按cDNA突变类型分为碱基重复27例（58.7％）、碱基缺失6例（13.0％）、碱基替换13例（28.3％）。其中23例（23/27，85.2％）表现为G重复突变（c.1927dupG，p.G642fs），4例（4/6，66.7％）表现为连续23个碱基缺失突变（c.1888_1910del:p.H630fs）。对三种突变类型患者的临床及实验室指标进行分析，结果显示碱基重复和碱基替换突变患者的中位异常细胞占有核细胞比例均明显高于碱基缺失突变患者（28％对17％，*P*＝0.047；48％对17％，*P*＝0.013）。

进一步用Mutation Taster软件对ASXL1三种碱基突变各位点进行致病性及保守性预测，结果示ASXL1碱基重复及缺失突变各位点均为致病性突变，替换突变各位点中有6例（6/13,46.2％）患者为致病性突变；碱基重复突变组的中位PhyloP值和PhastCons值均显著高于碱基缺失突变组（5.735对5.223，*P*<0.001；0.96对0.60，*P*＝0.012）（表2）。采用Alphafold2和Chimerax软件对蛋白结构进行预测及可视化，突变后相应蛋白的预测结构如图1所示，其结构域发生明显改变；用Chimera软件对代表性突变位点（c.1888_1910del:p.H630fs、c.1927dupG，p.G642fs、c.C3692T:p.S1231F）进行突变后蛋白结构差异性分析，结果显示与天然蛋白相比，碱基重复及替换突变RMSD值均高于缺失突变（3.25对2.98，3.07对2.98）。另外，对氨基酸的突变类型（移码、无义、错义）和氨基酸突变位点（p.G642fs突变组和非突变组）分别进行统计分析，以上氨基酸突变类型及突变位点分组中各亚组的临床、实验室指标及预后情况差异均无统计学意义（*P*值均>0.05）。

**表2:** 三种碱基突变类型突变位点致病性及保守性预测结果

碱基突变类型	例数	PhyloP值［*M*（范围）］	PhastCons值［*M*（范围）］
重复	27	5.735（−0.494～5.735）	0.96（0～1）
替换	13	0.96（−0.193～5.274）	0.96（0～1）
缺失	6	5.223（−0.193～5.223）	0.60（0～1）

*P_1_*值		<0.001	0.012
*P*_2_值		1.000	0.966

注：*P*_1_：重复突变与缺失突变比较；*P*_2_：替换突变与缺失突变比较

**图1:**
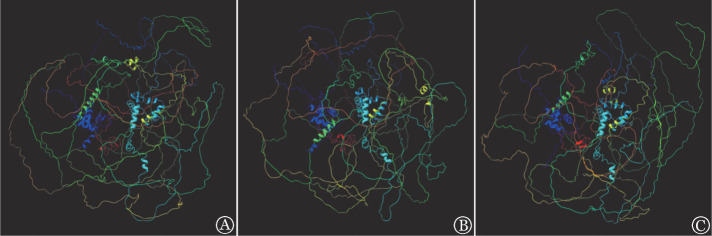
Alphafold2预测三种碱基突变类型的ASXL1蛋白结构图 A：碱基缺失突变（c.1888_1910del:p.H630fs）；B：碱基重复突变（c.1927dupG，p.G642fs）；C：碱基替换突变（c.C3692T:p.S1231F）

本研究中ASXL1突变的中位VAF为35.72％（1.07％～66.02％）。将患者根据VAF值分为<20％、20％～40％和≥40％ 三组，结果显示三组间外周血原始幼稚细胞比例差异有统计学意义（*P*＝0.037），其中高VAF组中位外周血原始幼稚细胞比例高于低VAF组（47.38％对21.21％，*P*＝0.029），其余临床及实验室指标差异均无统计学意义（*P*值均>0.05）。相关性分析结果显示，VAF值与初诊时年龄呈正相关（*P*＝0.043，*r*＝0.238）；与外周血原始幼稚细胞比例也呈正相关（*P*＝0.019，*r*＝0.301）。

将47例ASXL1^+^患者分为ASXL1单基因突变组和复合基因突变（≥2个基因突变，含ASXL1）组，比较两组临床及实验室指标，ASXL1单基因突变组骨髓原始细胞及异常细胞占有核细胞比例均高于复合基因突变组（*P*值分别为0.021和0.022）（表3）。37例复合基因突变患者突变情况见图2，进一步对基因突变的相关性分析，结果显示ASXL1突变常与IDH2（*P*＝0.018，*r*＝0.34）突变同时发生。

**表3:** ASXL1单基因突变与复合基因突变（含ASXL1基因）患者临床特征比较

临床特征	单基因突变组（10例）	复合基因突变组（37例）	统计量	*P*值
年龄［岁，*M*（范围）］	51（14~76）	59（18~85）	*z*＝−1.940	0.052
性别（例，男/女）	6/4	22/15	*χ*^2^＝0.000	1.000
WBC［×10^9^/L，*M*（范围）］	1.85（0.83~75.98）	5.8（0.59~271.47）	*z*＝−1.495	0.135
HGB［g/L，*M*（范围）］	76（21~132）	68（46~135）	*z*＝−0.208	0.835
PLT［×10^9^/L，*M*（范围）］	58（9~225）	39（2~298）	*z*＝−1.092	0.275
外周血原始幼稚细胞比例［％，*M*（范围）］	11（0~86）	26（0~89）	*z*＝−0.067	0.947
骨髓原始细胞比例［％，*M*（范围）］	69（0~95）	41（1~93）	*z*＝−2.314	0.021
异常细胞占有核细胞比例［％，*M*（范围）］	85（22~88）	24（5~71）	*z*＝−2.287	0.022

**图2:**
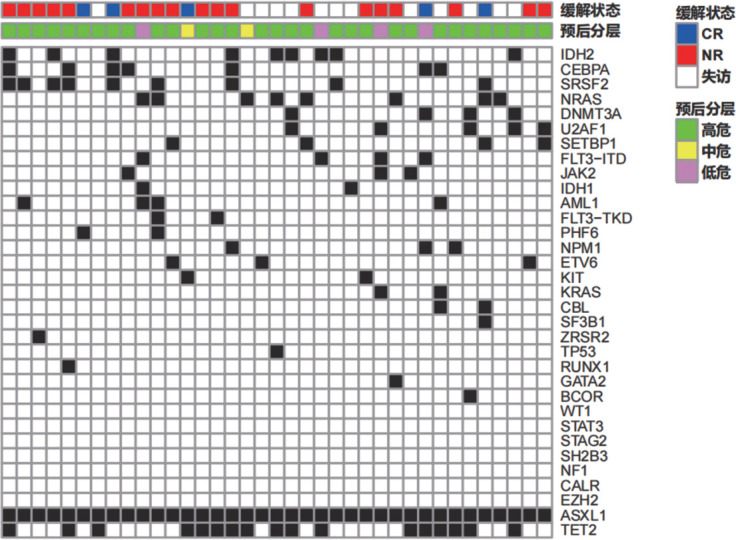
37例复合基因突变急性髓系白血病患者（≥2个基因突变，含ASXL1）的突变分布情况 CR：完全缓解；NR：未缓解

4. ASXL1突变患者的生存分析：231例未失访患者中，ASXL1^+^和ASXL1^−^患者中位OS时间分别为10（95％*CI* 1.38～18.62）个月和20（95％*CI* 14.54～25.47）个月（*P*＝0.012），中位PFS时间分别为10（95％*CI* 4.68～15.32）个月和 17（95％*CI* 13.43～20.57）个月（*P*＝0.002），前者明显短于后者（图3）。老年组中，ASXL1^+^与ASXL1^−^患者中位OS时间差异无统计学意义［6（95％*CI* 1.73～10.27）个月对11（95％*CI* 4.30～17.70）个月，*P*＝0.306］，而ASXL1^+^患者中位PFS时间为5（95％*CI* 0.84～9.16）个月，明显短于ASXL1^−^患者的9（95％*CI* 1.36～16.65）个月（*P*＝0.029）。中年组中，ASXL1^+^患者的中位OS和PFS时间均显著短于ASXL1^−^患者［OS时间：11（95％*CI* 0.61～21.40）个月对19（95％*CI* 13.58～24.42）个月，*P*＝0.033；PFS时间：10（95％*CI* 9.05～22.47）个月对16（95％*CI* 11.22～20.78）个月，*P*＝0.047］。青年组中，ASXL1^+^与ASXL1^−^患者间中位OS和PFS时间差异均无统计学意义［OS时间：28（95％*CI* 1.84～54.16）个月对37（95％*CI* 26.50～47.50）个月，*P*＝0.620；PFS时间：28（95％*CI* 1.84～54.16）个月对36（95％*CI* 25.05～46.14）个月，*P*＝0.520］。

**图3:**
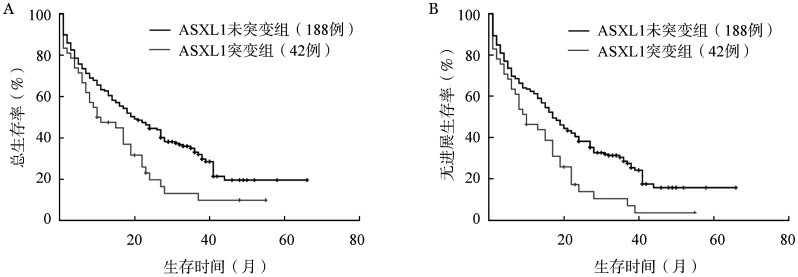
ASXL1^+^与ASXL1^−^急性髓系白血病患者总生存（A）和无进展生存（B）的比较

5. 预后影响因素分析：将性别、年龄、初诊时WBC、HGB、PLT、VAF、骨髓原始细胞比例、外周血原始幼稚细胞比例、异常细胞占有核细胞比例、复杂核型以及可能影响预后的基因突变等因素采用Cox比例风险回归模型的方法进行单因素预后分析（表4），进一步将*P*<0.05的因素纳入Cox多因素分析，结果表明异常细胞占有核细胞比例≥20％（*HR*＝2.678，95％*CI* 1.167～6.186，*P*＝0.020）、复杂核型（*HR*＝6.824，95％*CI* 1.180～39.458，*P*＝0.032）和TET2突变（*HR*＝2.441，95％*CI* 1.037～5.747，*P*＝0.041）均为影响ASXL1^+^患者OS的独立危险因素（表4）。

**表4:** 影响ASXL1突变急性髓系白血病患者总生存的单因素及多因素分析

临床特征	*HR*	95%*CI*	*P*值
单因素分析			
年龄（≥60岁/<60岁）	1.676	0.866~3.242	0.125
性别（男/女）	0.902	0.430~1.894	0.785
WBC（≥50×10^9^/L/<50×10^9^/L）	0.539	0.219~1.326	0.178
HGB（≥100 g/L/<100 g/L）	1.068	0.481~2.370	0.872
PLT（≥100×10^9^/L/<100×10^9^/L）	1.390	0.484~3.993	0.541
VAF（<20%/20%～40%/≥40%）	1.010	0.977~1.044	0.613
外周血原始幼稚细胞（≥20%/<20%）	0.852	0.429~1.692	0.647
骨髓原始细胞（≥80%/<80%）	1.189	0.556~2.545	0.655
异常细胞占有核细胞（≥20%/<20%）	2.152	1.067~4.339	0.032
复杂核型	6.800	1.319~35.05	0.022
FLT3-ITD突变	1.258	0.296~5.352	0.756
NPM1突变	0.547	0.074~4.048	0.555
CEBPA突变	1.296	0.534~3.146	0.567
TP53突变	6.154	0.758~49.948	0.089
RUNX1突变	1.926	0.736~5.036	0.182
DNMT3A突变	0.827	0.197~3.460	0.794
IDH2突变	0.781	0.318~1.198	0.590
SRSF2突变	0.981	0.439~2.195	0.964
TET2突变	2.239	1.087~4.611	0.029
多因素分析			
异常细胞占有核细胞（≥20%/<20%）	2.678	1.167~6.186	0.020
复杂核型	6.824	1.180~39.458	0.032
TET2突变	2.441	1.037~5.747	0.041

注：VAF：变异等位基因频率

## 讨论

ASXL1基因突变可促进造血干细胞（HSC）的克隆扩增，并导致造血功能受损[9]–[10]。此外，突变后的蛋白失去了与多梳抑制复合物2（PRC2）蛋白的相互作用，加速骨髓恶性肿瘤的进展，原因可能是野生型ASXL1能招募PRC2复合物到已知的白血病原癌基因位点，从而抑制疾病发生[9],[11]。另外，BAP1被证实是一种肿瘤抑制剂[12]，突变后的新等位基因可抑制野生型ASXL1与BAP1-TF的相互作用，从而损害ASXL1-BAP1-TF抑制白血病细胞增殖的功能[13]。以上可能是ASXL1突变致肿瘤发生的潜在机制。

有文献报道，伴ASXL1突变的AML患者高龄、男性较多[14]–[18]、外周血WBC较高[15]、外周血原始幼稚细胞比例和骨髓原始细胞比例较低[16]。本研究显示，ASXL1突变患者初诊时年龄及外周血WBC较高，与上述报道相似，但未发现与外周血原始幼稚细胞和骨髓原始细胞存在明显相关性，可能与研究病例数较少有关。

ASXL1突变与FLT3、NPM1、WT1、DNMT3A等突变呈负相关[14],[16],[18]，而常伴随TET2、IDH2、RUNX1、CEBPA突变[14],[16]–[18]。本研究中ASXL1突变与IDH2、TET2基因突变呈正相关，且与IDH2突变的相关性有统计学意义。有研究显示IDH2基因突变与AML的发生和CR/CR_i_降低有关[19]–[21]，提示ASXL1与IDH2可能是共同导致AML患者预后不良的基因。我们还发现ASXL1复合基因突变患者骨髓原始细胞、异常细胞占有核细胞比例较ASXL1单基因突变患者低，可能与复合基因突变患者伴较多预后好的突变基因有关，如NPM1等[22]–[23]，导致反映预后的指标总体好于ASXL1单基因突变患者。

在本研究的656例AML患者中，有11.1％发生ASXL1突变，与国外研究结果相仿[24]。Sasaki 等[25]研究表明ASXL1在AML患者中的中位VAF为34.31％，且VAF与新诊断AML患者的预后较差有关。本研究结果显示高突变率组中位外周血原始幼稚细胞比例高于低突变率组，且VAF与年龄和外周血原始幼稚细胞比例呈正相关关系，提示随着ASXL1基因VAF的升高，患者年龄、外周血原始幼稚细胞比例也随之变高，可能与预后不良有关，这与Sasaki等[25]的研究结果相似。

ASXL1基因突变在AML患者中主要表现为移码和错义突变，导致C端截断突变的ASXL1蛋白的产生[26]。已知碱基替换会导致错义突变，碱基重复及缺失突变均会导致移码突变。本研究首次发现碱基重复与替换突变患者中位异常细胞占有核细胞比例均高于碱基缺失突变患者，提示碱基缺失突变有较好预后，可用碱基突变位点的保守性和突变后蛋白结构间的差异性解释。首先，Mutation Taster软件预测碱基重复和缺失突变各位点为致病性突变，且重复突变反映保守性指标PhyloP和PhastCons值均高于缺失突变，值越高说明突变在生物体中发挥更大的作用，因此可认为碱基重复突变总体致病力强于缺失突变。另外，Chimera软件分析示碱基重复与替换突变后蛋白的RMSD值（反映蛋白质结构间差异性指标，值越高说明与天然蛋白差异越大）均高于缺失突变，说明前两种突变后蛋白结构的变化大于后者，失去更多正常功能，预后较差。

有文献报道伴ASXL1基因突变AML患者预后不良，表现为较短的OS时间[16],[18],[22],[24],[27]和PFS时间[24]和较低的CR/CR_1_率[21]，即使化疗后正常造血也难以恢复[28]。本研究中，ASXL1^+^患者OS及PFS时间均短于ASXL1^−^患者，与上述结果一致。其中，老年组ASXL1^+^患者PFS时间短于该基因未突变患者，而OS时间差异无统计学意义；中年组ASXL1^+^患者OS及PFS时间均短于ASXL1^−^患者。我们推测可能是年龄（≥60岁）对OS影响更大[29]，导致老年组中年龄因素相比ASXL1突变占据主导作用，致OS差异较小，而中年组中此作用相对降低，ASXL1突变对OS和PFS的影响得以显现。青年组ASXL1^+^与ASXL1^−^患者间OS与PFS时间差异无统计学意义，可能是青年组患者及其突变阳性病例数较少，影响该组生存分析结果的代表性。以上结果表明ASXL1基因突变为AML患者预后不良因素。

综上所述，伴ASXL1基因突变的非M_3_型AML患者，初诊时年龄偏高、WBC高、CR_1_率低，且OS、PFS时间短，预后较差。碱基缺失突变患者较其他突变患者可能有更好的预后，且异常细胞占有核细胞比例高、复杂核型和TET2突变为影响远期生存的危险因素，提示我们在临床治疗过程中应常规进行基因检测，以指导临床诊疗决策。碱基缺失突变相比其他突变能更好地反映预后，具体机制有待进一步探讨。
